# Screening and Validation of Functional Residues of the Antimicrobial Peptide *Pp*Rcys1

**DOI:** 10.3390/biom15111617

**Published:** 2025-11-18

**Authors:** Ming Tao, Zixun Fei, Aobo Sun, Guangming Yu, Huaiyuan Ye, Huishao Shi, Wei Zhang, Junjian Wang

**Affiliations:** 1 Biosafety Level 3 Laboratory, Medical School, Shenzhen University, Shenzhen 518060, China; tsmniu@szu.edu.cn; 2 College of Marine Science BGU, Beibu Gulf University, Qinzhou 535011, China; 3 College of Life Sciences and Oceanography, Shenzhen University, Shenzhen 518060, China

**Keywords:** antimicrobial peptides, *Pp*Rcys1, molecular dynamics simulations, structure–activity relationship

## Abstract

The excessive use of conventional antibiotics in aquaculture has created significant challenges, making it essential to explore and develop effective alternatives. Antimicrobial peptides (AMPs) have gained attention as potential therapeutic agents owing to their wide-ranging antibacterial effects and their ability to address pathogens resistant to conventional drugs. *Pp*Rcys1 is an antimicrobial peptide that mainly targets bacterial cell membranes, exhibiting a minimum inhibitory concentration of 8–32 μM. Its antibacterial activity should be further optimized. Before such optimization, however, it is crucial to identify the key amino acid residues that determine its functional activity. In this study, molecular dynamics simulations indicated that arginine 40 (ARG40), lysine 55 (LYS55), lysine 90 (LYS90), and lysine 93 (LYS93) play critical roles in the interaction between *Pp*Rcys1 and bacterial membranes. To investigate this further, these residues were mutated to serine, producing the mutant peptide *Pp*Rcys1_RMRK. Compared with *Pp*Rcys1, the mutant peptide *Pp*Rcys1_RMRK showed a significant reduction in antibacterial activity. Results from molecular dynamics simulations, Western blot, and ELISA demonstrated a marked decrease in its ability to bind to bacterial cell membranes. Membrane permeation assays, cell membrane depolarization experiments, and scanning electron microscopy revealed that *Pp*Rcys1 could not compromise the integrity of the bacterial membrane after losing ARG40, LYS55, LYS90 and LYS93. These findings highlight the critical roles of ARG40, LYS55, LYS90, and LYS93 in sustaining the antibacterial activity of *Pp*Rcys1. This study provides important initial insights into the structure–activity relationship of *Pp*Rcys1 and establishes a theoretical foundation for its future optimization.

## 1. Introduction

The problem of antibiotic overuse in aquaculture has become increasingly severe [1]. Beyond causing excessive drug residues in farmed aquatic products and threatening food safety, it also drives the emergence and spread of multidrug-resistant bacteria, thus reducing the effectiveness of traditional antibiotics [1,2]. This escalating resistance crisis complicates disease prevention and control in aquatic animals [1]. As a result, the search for alternative therapeutic agents that are safe, effective, and less likely to induce resistance has become an urgent priority. AMPs are usually small cationic molecules naturally present in innate immune systems. They exert their effects through multiple mechanisms, such as disrupting cell membranes, interfering with nucleic acid functions, and inhibiting protein synthesis. These mechanisms endow it with significant advantages, including wide-ranging antimicrobial effects, fast-acting pathogen elimination, and a reduced likelihood of promoting resistance development. Therefore, AMPs are regarded as promising candidates for new antibacterial drugs and have attracted widespread attention [3,4].

Although the Antimicrobial Peptide Database 3 (APD3) lists more than 3300 AMPs, only a small fraction have been applied in the prevention and control of aquaculture-related diseases [5]. This gap largely arises because many natural AMPs are prone to protease degradation, have limited structural stability or biocompatibility, and are costly to produce on a large scale [6,7]. To address these challenges, numerous studies have explored optimization and modification strategies aimed at improving the structural stability and antibacterial activity of AMPs while reducing their cytotoxicity [8,9]. For instance, Magainin-2 analogs exhibit a clear correlation between antimicrobial activity and net charge, where increasing positive charge enhances potency even when hydrophobicity and secondary structure are held constant [10]. Additionally, the deletion of the positively charged C-terminal region in melittin derivatives leads to a marked decrease in both hemolytic potential and membrane-binding capability [11].

Cationic residues in AMPs, such as lysine and arginine, are essential for mediating interactions with negatively charged bacterial cell membranes [12,13]. However, this feature also poses a considerable challenge: endogenous trypsin can selectively cleave at the C-terminal ends of the basic amino acids, markedly shortening the in vivo half-life of AMPs [14]. Hydrophobicity is another critical factor that requires careful balance. Hydrophobic residues, including alanine, leucine, and isoleucine, are predominantly positioned at the C-terminus of antimicrobial peptides. Their hydrophobic nature enables effective interactions with the nonpolar regions of bacterial cell membranes, facilitating membrane disruption and contributing to antimicrobial activity [15]. Excessive hydrophobicity can promote non-selective interactions with mammalian cell membranes, thus increasing hemolytic toxicity [16,17]. In contrast, insufficient hydrophobicity weakens membrane penetration and disruption, leading to reduced antibacterial activity [16].

To navigate the trade-offs among stability, selectivity, and antibacterial activity, researchers have focused on the structure–activity relationships of AMPs [9,18,19,20]. These studies have sought to clarify how key physicochemical properties—such as peptide structure, hydrophobicity, and amphiphilicity—correlate with functional outcomes, including antibacterial activity, hemolytic toxicity, cellular selectivity, and protease resistance [19]. Such insights provide a strong theoretical foundation for the rational design, optimization, and practical application of AMPs, thus supporting their advancement in fields such as aquaculture.

Molecular dynamics (MD) simulations computationally model the time-dependent behavior of molecular systems under defined conditions by numerically solving classical physical equations [21,22]. This method has been widely applied in AMP research [21,23,24]. For instance, MD simulations can predict and visualize how AMPs interact with negatively charged bacterial membranes, including their insertion into the lipid bilayer. They can also calculate the binding free energy of AMP–bacterial membrane interactions, thus clarifying the relationship between structural parameters and functional mechanisms of the peptides [25]. Previous studies have shown, for example, that changes in bilayer width can affect the dimerization of gramicidin within bacterial membranes, influencing its antimicrobial efficacy [26]. Similarly, Chen and Mark used MD simulations to explore how membrane curvature modulates different mechanisms of AMP activity [27].

*Pp*Rcys1 is a new cysteine-rich antimicrobial peptide from the *Pollicipes pollicipes*, and it demonstrates potent antibacterial activity [28]. Mechanistic studies indicate that its antimicrobial effect primarily arises from disruption of bacterial cell membranes [28]. Molecular dynamics simulations identified four key amino acid residues—ARG40, LYS55, LYS90, and LYS93—that are critical for mediating interactions between *Pp*Rcys1 and bacterial membranes. In this study, we engineered a mutant variant, *Pp*Rcys1_RMRK, in which these four residues were substituted with serine. The designation “_RMRK” is an abbreviation for “Remove R (Arginine) and K (Lysine)”. Theoretically, single-residue mutagenesis represents the classical approach for assessing the contribution of individual amino acid residues [29]. However, given the relatively high molecular weight of *Pp*Rcys1 and based on our prior experimental experience, we anticipate that mutating a single cationic residue may result in only subtle alterations in antibacterial activity. Such minor changes could fall below the detection sensitivity of the standard double dilution assay employed in this study, thereby limiting reliable quantification [30,31]. Consequently, simultaneous mutation of four key residues constitutes a more practical and effective strategy, as it is more likely to generate pronounced phenotypic differences that can be accurately measured. Should this multi-site mutagenesis yield meaningful insights, it would provide a robust foundation for subsequent, more targeted single-point mutagenesis studies. Comparative analysis of the *Pp*Rcys1 and *Pp*Rcys1_RMRK by laboratory experiments validated the predictions of the MD simulations and provided new insights into the structure–activity relationship of *Pp*Rcys1. These results provide a scientific basis for the further refinement and development of *Pp*Rcys1 in future studies.

## 2. Materials and Methods

### 2.1. Bacterial Strains and Growth Conditions

To compare the differences in antibacterial activity between PpRcys1 and its mutants, the strains used in this study were consistent with those in previous studies [28]. This study employed seven bacterial species, including three Gram-positive strains, *Bacillus* sp. T2, *Staphylococcus aureus* (ATCC 6538), and *Streptococcus agalactiae* (ATCC 13813), and four Gram-negative strains, *Vibrio alginolyticus* (ATCC 17749), *Escherichia coli* (ATCC 25922), *Aeromonas hydrophila* (ATCC 35654), and *Acinetobacter* sp. L32. All strains were preserved as glycerol stocks at −80 °C and are maintained in the Shellfish Breeding Laboratory at Beibu Gulf University [32]. Prior to experimentation, each strain was inoculated into 2 mL of the appropriate culture medium and incubated at 37 °C with shaking at 200 rpm for 12 h to ensure optimal growth. For culture purposes, *S. agalactiae*, *S. aureus*, *E. coli*, *A. hydrophila*, and *Acinetobacter* L32 were grown in Luria–Bertani (LB) broth (ST163, Beyotime, Shanghai, China), while V. alginolyticus was grown in Zobell Marine Broth 2216 (2216E) medium (HB0132, Haibo, Qingdao, Shandong, China).

### 2.2. MD Simulations

A heterogeneous membrane model was constructed using the Membrane Builder module in CHARMM-GUI [33]. The final membrane system had a surface area of 12 × 12 nm^2^, with each monolayer composed of 366 POPE and 122 POPG lipid molecules, yielding a POPE-to-POPG molar ratio of 3:1 [34,35]. T The three-dimensional structure of *Pp*Rcys1 was predicted using AlphaFold2. At physiological pH (7.4), all ionizable residues of *Pp*Rcys1 were assigned their canonical protonation states. The protein structure was then translated along the membrane normal (z-axis) to a position 50 Å above the bilayer surface. The CHARMM36 force field was employed to parameterize both the lipid bilayer and the *Pp*Rcys1 peptide, while the TIP3P water model was used for solvation [36]. Solvation boxes with dimensions of 12 × 12 × 16 nm^3^ were generated, and ionic neutrality was maintained by adding Na^+^ and Cl^−^ ions to each system. All simulations were performed using GROMACS version 2023.3 [37], and trajectory analysis was conducted using VMD version 1.9.3 [38].

Molecular dynamics simulations were carried out using GROMACS 2023.3. Following energy minimization and equilibration in both NVT and NPT ensembles, three independent production runs were conducted, each lasting 300 nanoseconds. Simulation parameters were as follows: energy minimization was performed using the steepest descent algorithm with a convergence threshold of 1000 kJ·mol^−1^·nm^−1^. This was followed by a 500 ps equilibration phase under NVT and NPT conditions. Temperature was maintained at 310 K using the V-rescale thermostat [39], and pressure was regulated at 1 bar using the C-rescale barostat [40]. Long-range van der Waals interactions were truncated at a cutoff distance of 1.0 nm. Electrostatic interactions were computed using the particle-mesh Ewald (PME) method [41]. A time step of 2 fs was used during production runs, with trajectories saved every 10 ps. Trajectory snapshots were visualized using VMD [38] and periodic boundary conditions were applied in all three spatial dimensions (x, y, z) [28]. Trajectory data were recorded at an interval of one frame per 100 ps. Root mean square deviation (RMSD) of the protein backbone atoms was calculated over time to assess conformational stability. Furthermore, the MM-PBSA method was employed to identify key functional residues involved in membrane binding by evaluating residue-wise free energy contributions based on simulation data collected after the system reached equilibrium—defined by the stabilization of cumulative averages of electrostatic and van der Waals interaction energies [42]. This analysis revealed that residues ARG40, LYS55, LYS90, and LYS93 play critical roles in mediating the initial adsorption of *Pp*Rcys1 to the bacterial membrane. Based on these findings, these residues were substituted with serine to generate a mutant designated *Pp*Rcys1_RMRK, and its three-dimensional structure was subsequently predicted using AlphaFold2 [43].

### 2.3. Heterologous Expression and Purification of Recombina PpRcys1_RMRK (rPpRcys1_RMRK)

In our previous study, *Pp*Rcys1 was fused with the His-SUMO tag. The His-SUMO-PpRcys1 protein was obtained using *E*. *coli* BL21 (DE3), and the His-SUMO tag was then excised using the SUMO enzyme to obtain recombinant *Pp*Rcys1 (r*Pp*Rcys1) [28]. Based on previous studies, both r*Pp*Rcys1 and His-SUMO-*Pp*Rcys1 proteins have been successfully expressed [28]. Binding free energy analysis identified residues ARG40, LYS55, LYS90, and LYS93 as crucial for mediating the initial adsorption of PpRcys1 to the bacterial membrane. Building on these findings, each of these residues was replaced with serine to generate the mutant *Pp*Rcys1_RMRK. To obtain the mutant, *Pp*Rcys1_RMRK, the codons encoding ARG40, LYS55, LYS90, and LYS93 in the *Pp*Rcys1 coding sequence were replaced with serine codons. The *Pp*Rcys1_RMRK coding sequence was chemically synthesized by General Biosystems company and flanked at both ends with BamHI and XhoI restriction sites (Chuzhou, China). The synthesized gene fragment was inserted into the pSmartI vector, which carries a His-SUMO tag, using BamHI and XhoI restriction enzyme digestion, resulting in the recombinant plasmid pSmartI-*Pp*Rcys1_RMRK (5814 bp), as illustrated in Supplementary Figure S1. The recombinant protein was expressed in *E. coli* BL21 (DE3) cells, and positive transformants were verified by PCR amplification and DNA sequencing prior to protein expression. Detailed information on primer design and PCR cycling parameters is provided in Supplementary Tables S1 and S2. Protein expression was initiated by supplementing the culture with isopropyl β-D-thiogalactoside (IPTG) to achieve a final concentration of 0.5 mM. Following induction, the bacterial culture was maintained at 16 °C under continuous agitation for 12 h. The cells were collected and disrupted with E. coli lysis buffer, then centrifuged at 10,000 rpm for 30 min at 4 °C to isolate the soluble fraction from insoluble cellular material. Protein expression levels were assessed by sodium dodecyl sulfate–polyacrylamide gel electrophoresis (SDS–PAGE) of both induced and non-induced whole-cell lysates. The His-SUMO-*Pp*Rcys1_RMRK fusion protein in the supernatant was purified using Ni-NTA affinity chromatography and subsequently dialyzed in 1× PBS buffer at 4 °C for 24 h. To remove the His-SUMO tag, the fusion protein was treated with one unit of SUMO protease (General Biosystems, Chuzhou, Anhui, China) and incubated at 4 °C for 6 h. During a subsequent Ni-column purification, the His-SUMO tag remained bound to the resin, while the cleaved, untagged recombinant *Pp*Rcys1_RMRK (r*Pp*Rcys1_RMRK) was collected in the flow-through fraction. The purity of the final protein preparation was evaluated by SDS–PAGE, and protein concentration was measured using a BCA assay kit (Beyotime, Shanghai, China) following the manufacturer’s protocol. Subsequently, the purified protein was lyophilized and kept at −80 °C for long-term storage and subsequent applications.

Following the procedure described in our previous study [28], SDS–PAGE bands corresponding to r*Pp*Rcys1*_RMRK* were carefully excised and placed into microcentrifuge tubes. To facilitate destaining, we added 50% (*v*/*v*) acetonitrile, and the samples were incubated with shaking at 37 °C overnight. Pure acetonitrile was then introduced to promote precipitation of the protein, which was subsequently removed. The proteins were digested with trypsin in ammonium bicarbonate buffer (Tianjingsha Gene Technology Co., Ltd., Beijing, China) and incubated in a 37 °C water bath for 16 h. Following digestion, the solution was transferred to a fresh tube and mixed with an extraction solvent consisting of water and anhydrous acetonitrile (1:4, *v*/*v*), then acidified with 0.5% formic acid. The mixture was processed through ultrasonication, centrifugation, and vacuum concentration to yield dried protein samples. Before analysis, the residue was reconstituted in a solvent system of water and anhydrous acetonitrile (1:49, *v*/*v*) containing 0.5% formic acid, and homogenized thoroughly via vortexing and shaking. The resulting samples were then subjected to LC–MS analysis using a TRIPLETOF 5600+ system (ABSCIEX, Framingham, MA, USA) for protein identification.

### 2.4. Analysis of Physical and Chemical Properties and Structure

Using the predictive tools of the APD3 database, the physicochemical properties of *Pp*Rcys1 and *Pp*Rcys1_RMRK were analyzed, including their molecular weight, isoelectric point, net charge, Wimley–White whole-residue hydrophobicity, grand average of hydropathy (GRAVY), and Boman index [5]. Three-dimensional models of both peptides were subsequently constructed using AlphaFold2 [43]. The predicted Local Distance Difference Test (pLDDT) results of AlphaFold2 are shown in Supplementary Figure S2.

### 2.5. Assay of Antimicrobial Activities of rPpRcys and rPpRcys1_RMRK

The minimum inhibitory concentrations (MICs) of r*Pp*Rcys1 and r*Pp*Rcys1_RMRK were assessed using a modified microtiter plate method for antimicrobial susceptibility, in accordance with the Clinical and Laboratory Standards Institute (CLSI) guidelines. Bacterial cultures were cultivated to an OD600 of 0.4 and subsequently diluted to a final concentration of 10^4^ CFU/mL in Mueller–Hinton broth (MHB; HB6232, Haibo, Qingdao, China). Each peptide, r*Pp*Rcys1 and r*Pp*Rcys1_RMRK was individually dissolved in 1× PBS. In the assay, 20 μL of peptide solution was combined with 80 μL of the bacterial suspension in the wells of a 96-well microplate. Serial dilutions of the peptides were tested at the following concentrations: 64, 32, 16, 8, 4, 2, and 1 μM. Ampicillin served as the positive control, and 1× PBS was used as the negative control. Microplates were incubated at 37 °C for 18 h [28]. The MIC was defined as the lowest peptide concentration that completely inhibited bacterial growth, as determined using a resazurin-based viability assay with OD560 and OD590 measurements [29]. Growth curves of *S. aureu* and *V. alginolyticus* were subsequently constructed by measuring OD600 at 0, 4, 8, 12, 24, and 48 h at a peptide concentration of 64 μM, using BSA as control proteins. All experiments were conducted in triplicate, incorporating both biological and technical repetitions, to guarantee consistency and reproducibility of the results.

### 2.6. Binding Assay for Membrane Mimetic

To optimize the lipid formulation, a molar ratio of 7.5:2.5 nmol of 1-palmitoyl-2-oleoylphosphatidylethanolamine (POPE) to phosphatidylglycerol (POPG) was chosen, yielding a total lipid concentration of 100 μM. The lipids were first dissolved in chloroform, after which the solvent was evaporated under a gentle stream of nitrogen gas. The lipid film was further dried under high vacuum for 1 h to eliminate any residual organic solvent completely. Liposome formation was achieved by hydrating the dried lipid film with preheated HEPES buffer (20 mM HEPES, 150 mM NaCl, pH 7.4). The resulting dispersion was vortexed and subsequently sonicated to generate small unilamellar vesicles—either through probe sonication using 10 cycles of 10 s pulses on ice or via bath sonication at 55 °C for 30 min. The liposomal suspension was then diluted to a working concentration of 10–20 μg/mL and added to a 96-well microplate at 100 μL per well. Plates were incubated overnight at 4 °C to allow liposomes to adsorb onto the well surfaces. After adsorption, each well was washed three times with 1× phosphate-buffered saline containing 0.05% Tween 20 (PBST, pH 7.4, 60146ES76, Yeasen, Shanghai, China). To minimize non-specific binding, 100 μL of blocking solution (5% skim milk in 1× PBST, pH 7.4) was added to each well, followed by incubation at 37 °C for 2 h. Finally, the wells were gently rinsed three times with 1× PBST to remove excess blocking agent before further use.

Since there are no tags in r*Pp*Rcys1 and r*Pp*Rcys1_RMRK, they cannot be directly used for the binding activity test. Therefore, His-Sumo-pprcys1_RMRK and HIS-SUmo-PPRcYS1 proteins were used for the binding activity test, with the His-SUMO tag serving as the control. His-SUMO-*Pp*Rcys1_RMRK was diluted to a concentration of 10 μM in 1× PBS (pH 7.4) and added to the corresponding wells. As a positive control, 10 μM bovine serum albumin (BSA) was applied, while 10 μM of the His-SUMO tag alone was used as the negative control for comparison. The plate was incubated at 37 °C for 1 h, followed by a single wash with 1× PBST (pH 7.4). Subsequently, 100 μL of horseradish peroxidase (HRP)-labeled anti-His antibody, diluted 1:5000 in 1× PBST (pH 7.4; Boyi, Changzhou, China), was added to each well. The plate was incubated at 37 °C for 1 h, followed by five washes with 1× PBST to eliminate unbound antibody. To detect binding, 100 μL of TMB substrate solution was added to each well to trigger colorimetric development. The reaction was terminated promptly by adding 200 μL of ELISA stop solution per well. The absorbance was then read at 450 nm using a microplate reader (Synergy™ LX, BioTek, Kaysville, UT, USA). The binding assay for His-SUMO-*Pp*Rcys1 to bacterial membrane mimics was performed using the same procedure as for His-SUMO*-Pp*Rcys1. To ensure reproducibility and reliability, the experiment was conducted with three biological replicates and three technical replicates.

### 2.7. Microorganism-Binding Assay

The experimental protocol of microorganism-binding assay was performed, as described previously for *Pp*Rcys1 [28]. Briefly, 1 × 10^8^ CFU of *S. aureus* was transferred to 1.5 mL centrifuge tubes and incubated with 200 μL of 5 μM His-SUMO-*Pp*Rcys1 or His-SUMO-*Pp*Rcys1_RMRK for 1 h at 30 °C with gentle rotation. After incubation, bacterial cells were collected, washed three times with 1× TBS, and resuspended. After centrifugation at 10,000 rpm for 5 min, the resulting cell pellets were analyzed by SDS–PAGE to evaluate protein binding. Proteins were electrophoretically transferred onto a polyvinylidene fluoride (PVDF) membrane, which was then blocked with 5% skim milk in 1× TBST. The membrane was incubated with HRP-conjugated anti-His antibody diluted at 1:30,000 (Boyi, Changzhou, China). Protein bands were detected using BeyoECL Plus (Beyotime, Shanghai, China) and visualized with a chemiluminescent imaging system (WD-9423B/C, Liuyi, Beijing, China) according to the manufacturer’s protocol, using a 10 s exposure time. The intensity of protein bands was analyzed and quantified using ImageJ v1.54r software.

### 2.8. Membrane Permeability Assay

The membrane-disruptive activities of *Pp*Rcys1_RMRK and *Pp*Rcys1 against *S. aureus* and *V. alginolyticus* were assessed using a lactate dehydrogenase (LDH) release assay. Bacterial cells were collected during the mid-logarithmic growth phase (OD600 ≈ 0.5), washed, and resuspended in phosphate-buffered saline (PBS). A volume of 100 μL of bacterial suspension was transferred into a 96-well plate and treated with either 64 μM PpRcys1_RMRK or PpRcys1 for 2 h. Following incubation, samples were centrifuged at 12,000× *g* for 2 min. Then, 50 μL of the supernatant was combined with 50 μL of a reaction mixture consisting of 50 mM sodium phosphate buffer (pH 7.5), 0.6 mM pyruvate, and 0.2 mM NADH. The reaction was allowed to proceed at room temperature for 10 min and then stopped by adding 50 μL of 1 M acetic acid. Absorbance was measured at 340 nm using a Tecan Spark plate reader (Männedorf, Switzerland). To measure total LDH content, bacterial cells lysed with 1% Triton X-100 were used as the control [44,45]. The percentage of LDH release, reflecting membrane disruption by r*Pp*Rcys1 and r*Pp*Rcys1_RMRK, was calculated as the ratio of LDH activity in treated samples to that in fully lysed cells using the following formula:

$$\mathrm{Permeability}\;(\%)\;=\;\frac{OD340\left(Sample\right)-OD340(BSA)}{OD340\left(TritonX-100treated\right)-OD340(BSA)}\;\times \;100\%$$

Controls included bacteria treated by1% Triton X-100 (positive) and BSA (negative).

### 2.9. Assessment of Membrane Depolarization

In several instances, disruption of the membrane potential serves as the primary mechanism of action or enhances the effectiveness of a compound. This effect may result from the formation of ion-conducting pores in the membrane, a general increase in membrane ion permeability, or the molecule functioning as an ion shuttle [46,47,48]. Consequently, evaluating membrane permeability is a key experimental step in determining the mode of action of membrane-targeting AMPs. To assess the membrane depolarization effects of r*Pp*Rcys1 and r*Pp*Rcys1_RMRK, we used the membrane potential-sensitive fluorescent dye 3,3′-dipropylthiacarbocyanine iodide (DiSC3-5; Sigma–Aldrich, St. Louis, MO, USA) following a previously described method [49]. Bacterial cells in the mid-logarithmic growth phase were harvested by centrifugation at 6000× *g* for 5 min. The resulting pellet was washed with a buffer composed of 5 mM HEPES (pH 7.3) and 20 mM glucose, then resuspended in the same buffer containing an additional 100 mM KCl. A 100 μL aliquot of bacterial suspension (OD600 = 0.05) was combined with 0.5 μM DiSC3-5 and transferred to a 96-well white microplate (2070110, SAINING, China). The plate was incubated for 30 min to allow the fluorescence signal to stabilize. Subsequently, 100 μL of protein solution—r*Pp*Rcys1, r*Pp*Rcys1_RMRK, or BSA—was added to each well to achieve a final concentration of 64 μM, with BSA serving as the control. Fluorescence intensity was recorded continuously over a 15 min period using a TECAN GENios Plus spectrofluorometer (Männedorf, Switzerland), with excitation set at 622 nm and emission measured at 670 nm.

### 2.10. Scanning Electron Microscopy (SEM)

SEM was performed following previously published methods [28] *V. alginolyticus* and *S. aureus* were cultured in LB and 2216E media, respectively, until reaching the mid-logarithmic growth phase. Bacterial cells were harvested and resuspended in 1× PBS to achieve a final concentration of 10^6^ CFU/mL. The suspensions were incubated with 64 μM r*Pp*Rcys1_RMRK for 2 h on round coverslips positioned in 24-well plates. Following incubation, samples were fixed overnight at 4 °C using 5% glutaraldehyde in PBS (pH 7.4), then washed three times with 1× PBS. Bacterial cells treated with BSA were used as the control. Dehydration was performed using a stepwise ethanol series (30, 50, 70, 80, 90, and 100%) at 4 °C, with each step lasting 10 min. Samples were then processed using a critical point dryer (Hitachi-HCP, Hitachi, Tokyo, Japan), coated with a thin layer of gold via sputtering (MC1000, Hitachi, Tokyo, Japan), and examined under a scanning electron microscope (APREO S, Thermo Fisher Scientific, Waltham, MA, USA).

### 2.11. Statistical Analysis

Data analysis was conducted using GraphPad Prism 10.0 (GraphPad, San Diego, CA, USA). One-way analysis of variance (ANOVA) was applied to evaluate statistical significance, and all results are presented as mean values ± standard deviation (SD). A *p*-value less than 0.05 was regarded as statistically significant.

## 3. Results

### 3.1. Comparison of the Sequences and Structures of PpRcys1_RMRK and PpRcys1

**Figure 1:**
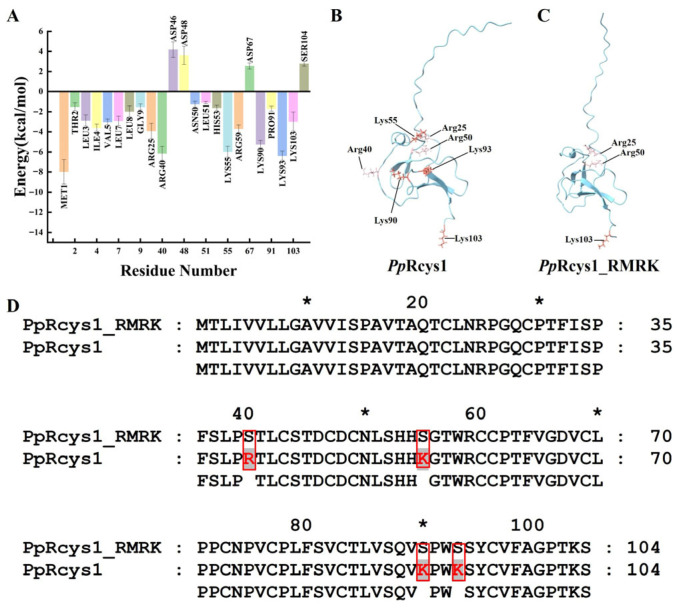
Binding free energy analysis of *Pp*Rcys1 and comparative analysis between *Pp*Rcys1 and *Pp*Rcys1_RMRK. (**A**) Binding free energy analysis of each amino acid in *Pp*Rcys1. (**B**,**C**) The three-dimensional structures of *Pp*Rcys1 and *Pp*Rcys1_RMRK, respectively. Lysine is marked in red, and arginine is in pink. (**D**) Sequence alignments of *Pp*Rcys1 and *Pp*Rcys1_RMRK. The mutation sites are marked with red boxes. * indicates that this is the position of the 10th, 30th, 50th, 70th and 90th amino acids.

Based on the previous work, we have obtained the conformation set of *Pp*Rcys1 through simulation in solution (Supplementary Figure S3) and selected representative conformations for testing in a membrane environment [28]. Three independent 300 ns molecular dynamics simulations of *Pp*Rcys1 were conducted. Analysis indicates that both electrostatic and van der Waals interactions stabilize after approximately 275 ns, suggesting that the system has reached equilibrium. As a result, the binding free energy was calculated using the final 25 ns (275–300 ns) of the simulation trajectories to ensure convergence (Supplementary Figure S4). During this period, *Pp*Rcys1 remains stably adsorbed on the membrane surface, maintaining persistent intermolecular interactions. Combined with free energy analysis, key amino acid residues involved in the adsorption of antimicrobial peptides onto bacterial cell membranes can be identified. Analysis of the binding free energies by MMPBSA revealed that ARG40, LYS55, LYS90, and LYS93 were −6.13, −5.97, −5.22, and −6.37 kcal/mol, respectively, indicating that these residues favored the adsorption of *Pp*Rcys1 onto the bacterial cell membrane. In contrast, ASP46, ASP48, ASP67, and SER104 exhibit binding free energies greater than 2.5 kcal/mol, which hinder membrane adsorption of *Pp*Rcys1 (Figure 1A).

Based on these results, ARG40, LYS55, LYS90, and LYS93 were mutated to serine, generating the mutant peptide *Pp*Rcys1_RMRK (Figure 1B,C). The three-dimensional models of *Pp*Rcys1 and *Pp*Rcys1_RMRK were established by using Alphafold2. The analysis indicated that *Pp*Rcys1_RMRK retains a CSαβ-fold, one β-sheet, and several coil regions. The coding sequence (CDS) of *Pp*Rcys1_RMRK spans 312 bp, of which 255 bp encode the mature peptide. Furthermore, the protein features an N-terminal signal peptide comprising amino acids 1 to 19.

### 3.2. Comparison of the Physical and Chemical Properties of PpRcys1_RMRK and PpRcys1

Compared with *Pp*Rcys1, *Pp*Rcy1s_RMRK exhibited a significantly reduced net charge (decreased by 88.89%), and its isoelectric point decreased from 8.50 to 6.65, representing a 21.76% reduction. The Boman index and Wimley–White whole-residue hydrophobicity of *Pp*Rcys1_RMRK decreased by 53.13% and 107.59%, respectively, indicating a substantial reduction in hydrophobic properties. In contrast, the grand average of hydropathy (GRAVY) for *Pp*Rcys1_RMRK increased by 25% compared to *Pp*Rcys1, suggesting enhanced hydrophilicity. The molecular weight of *Pp*Rcys1_RMRK was only slightly reduced by 1.79% relative to that of *Pp*Rcys1 (see Table 1).

### 3.3. Recombinant Expression, Purification, and Identification of PpRcys_RMRK

**Figure 2:**
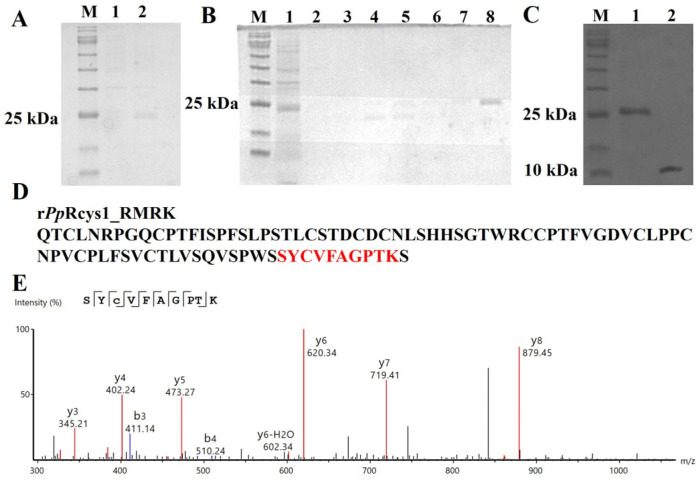
Acquisition and MS spectral analysis of r*Pp*Rcys1_RMRK. (**A**) SDS–PAGE analysis of recombinant *Pp*Rcys1_RMRK (r*Pp*Rcys1_RMRK) expressed in E. coli with a His-SUMO tag. Lane M represents the protein molecular weight marker; lane 1 displays total protein from non-induced E. coli, and lane 2 shows total protein following IPTG induction. (**B**) Purification of His-SUMO-*Pp*Rcys1_RMRK using Ni-NTA affinity chromatography. Lane M: protein marker; lane 1: unbound proteins; lane 2: equilibration buffer; lanes 3–8: elution fractions containing 20, 50, 100, 150, 300, and 500 mM imidazole, respectively. (**C**) SDS–PAGE analysis of r*Pp*Rcys1_RMRK after removal of the SUMO tag. Lane M: protein marker; lane 1: His-SUMO-*Pp*Rcys1_RMRK prior to SUMO protease treatment; lane 2: purified tag-free r*Pp*Rcys1_RMRK. (**D**) Sequence alignment between mass spectrometry data and the theoretical rRcys1_RMRK sequence. (**E**) LC–MS spectrum of the peptide “SYCVFAGPTK”, where the red, blue, and black traces represent y-type ions, b-type ions, and background noise signals detected by mass spectrometry, respectively.

The heterologous expression, purification, and identification of *Pp*Rcys1_RMRK were performed following the same procedures as for *Pp*Rcys1. SDS–PAGE analysis revealed clear differences in protein banding patterns before and after IPTG induction, showing a prominent band at approximately 25 kDa (Figure 2A). This band aligns with the expected molecular weight of the His-SUMO-*Pp*Rcys1_RMRK fusion protein, which consists of the His-SUMO tag (~18 kDa) and the mature *Pp*Rcys1_RMRK peptide (9.16 kDa). The fusion protein was effectively purified using Ni-NTA affinity chromatography with gradient imidazole elution, achieving optimal elution at 500 μM imidazole (Figure 2B, lane 8). Subsequent digestion with SUMO protease successfully generated the tag-free recombinant *Pp*Rcys1_RMRK (r*Pp*Rcys1_RMRK), which was identified as a ~10 kDa protein by SDS–PAGE.

LC–MS analysis was performed to verify the amino acid composition of r*Pp*Rcys1_RMRK, resulting in the detection of a single peptide that provided 11.76% sequence coverage (Figure 2D,E). Notably, the sequence coverage calculation was restricted to the region corresponding to the mature peptide.

### 3.4. Comparison of the Antibacterial Activities of rPpRcys1_RMRK and rPpRcys1

**Figure 3:**
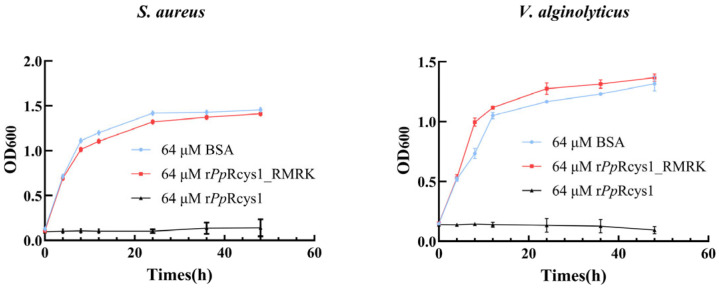
The influence of r*Pp*Rcys1_RMRK and r*Pp*Rcys1 on the growth rate of *S. aureus* and *V. alginolyticys*. Bacterial growth curves were generated by measuring OD600 at time points of 0, 4, 8, 12, 24, and 48 h in the presence of 64 μM peptide. The experiment was performed with three biological replicates, each consisting of three technical replicates, and BSA was used as the control.

The minimum inhibitory concentrations (MICs) of r*Pp*Rcys1 and r*Pp*Rcys1_RMRK, were determined using a modified microtiter plate-based antimicrobial susceptibility assay following CLSI guidelines (Table 2). Compared with r*Pp*Rcys1, the antibacterial activity of r*Pp*Rcys1_RMRK was significantly reduced. At a concentration of 64 μM, r*Pp*Rcys1_RMRK showed no inhibitory effect on any strain. At the same concentration, the effect of r*Pp*Rcys1_RMRK on bacterial growth rate was not significantly different from that of BSA (Figure 3).

### 3.5. Comparison of Membrane- and Microorganism-Binding Activities Between rPpRcys1_RMRK and rPpRcys1

**Figure 4:**
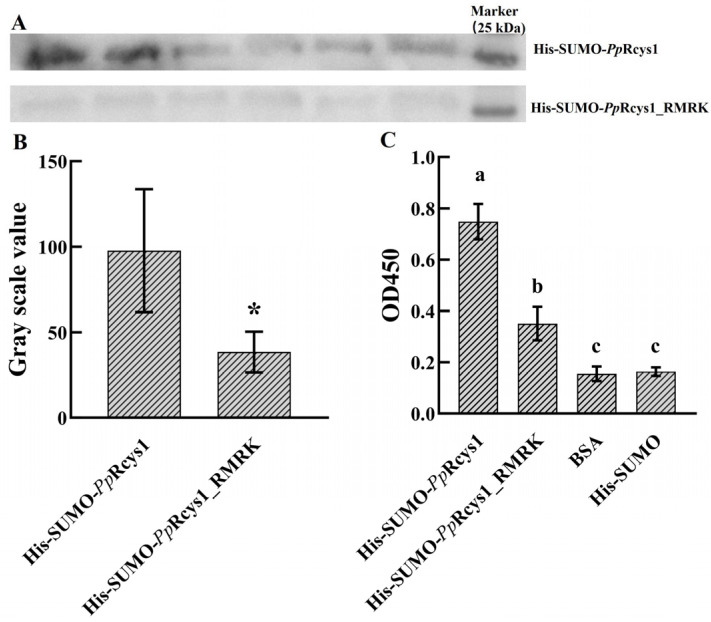
Microorganism and PAMP-binding activities of r*Pp*Rcys1 and r*Pp*Rcys1_RMRK. (**A**) Assay of Microorganism-binding activity. His-SUMO-*Pp*Rcys1 and His-SUMO-*Pp*Rcys1_RMRK were detected by Western blot after treatment with *S. aureus*. Upper panel: His-SUMO-*Pp*Rcys1; lower panel: His-SUMO-*Pp*Rcys1_RMRK. (**B**) Quantification of the Western blot bands. * indicates a significant difference compared with the His-SUMO-*Pp*Rcys1 at *p* < 0.05. The gray value (Y-axis) represents the gray band intensity in Figure 5A, with higher values indicating stronger binding ability with Staphylococcus aureus. (**C**) Membrane mimic binding assay. BSA was used as the control, while the His-SUMO tag was employed as the negative control. The experiments were conducted using three biological replicates, each with three technical replicates. Distinct lowercase letters (a, b, c) denote statistically significant differences at * *p* < 0.05, while groups labeled with the same letter show no significant difference (* *p* > 0.05).

In the Western blot experiment, the average gray value of the His-SUMO-*Pp*Rcys1 band was 97.83, whereas that of the His-SUMO-*Pp*Rcys1_RMRK band was 38.5, representing a 60.64% decrease compared to His-SUMO-*Pp*Rcys1 (Figure 4A,B). Consistently, the binding ability of His-SUMO-*Pp*Rcys1_RMRK to bacterial membrane mimics was significantly lower than that of His-SUMO-*Pp*Rcys1 (Figure 4C). These results indicate that, compared with r*Pp*Rcys1, the bacterial binding activity of r*Pp*Rcys1_RMRK is markedly reduced.

### 3.6. Comparison of the Effects of rPpRcys1_RMRK and rPpRcys1 on Membrane Depolarization and Membrane Permeability

**Figure 5:**
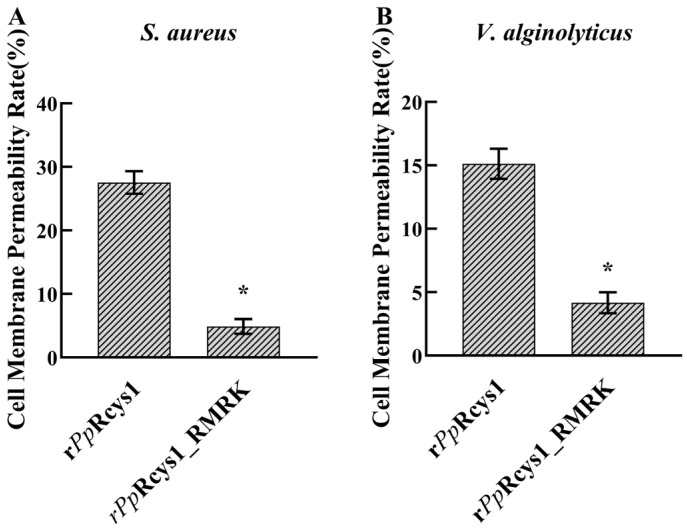
Impact of r*Pp*Rcys1_RMRK and r*Pp*Rcys1 on membrane permeability in *S. aureus* (**A**) and *V. alginolyticus* (**B**). Each assay was performed with three biological replicates, and each replicate included three technical repetitions. *, denotes a statistically significant difference relative to the r*Pp*Rcys1 treatment at * *p* < 0.05.

**Figure 6:**
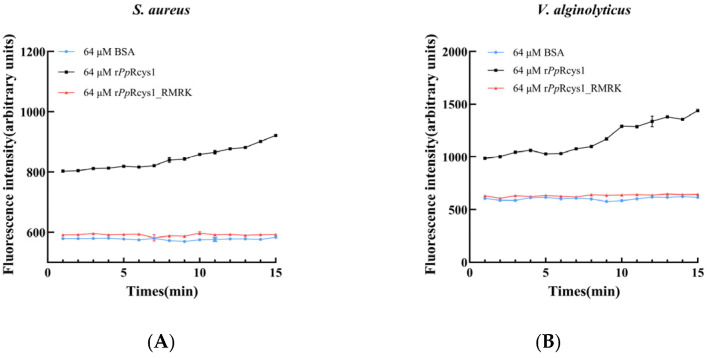
Effects of r*Pp*Rcys1_RMRK and r*Pp*Rcys1 on bacterial membrane depolarization of *S. aureus* (**A**) and *V. alginolyticus* (**B**). Bacterial membrane depolarization was assessed using DiSC3-5, with BSA serving as the control. The experiments were conducted in triplicate (three biological replicates), each comprising three technical replicates.

The release of intracellular lactate dehydrogenase (LDH) occurs when microbial membrane integrity is compromised, making it a marker for bacterial cell membrane permeability. After treatment with 64 μM r*Pp*Rcys1 for 2 h, the membrane permeabilities of *V. alginolyticus* and *S. aureus* were 15.11 and 27.53%, respectively (Figure 5A). In contrast, treatment with 64 μM r*Pp*Rcys1_RMRK for 2 h resulted in much lower membrane permeabilities of 4.88% for *S. aureus* and 4.17% for *V. alginolyticus* (Figure 5B).The membrane depolarization activities of r*Pp*Rcys1_RMRK and r*Pp*Rcys1 were evaluated using the potential-sensitive fluorescent dye DiSC3-5. Compared with the BSA control, treatment with r*Pp*Rcys1 significantly increased the fluorescence intensities of *V. alginolyticus* and *S. aureus* (Figure 6), whereas r*Pp*Rcys1_RMRK did not induce a similar increase. These results indicate that r*Pp*Rcys1 can trigger bacterial plasma membrane depolarization, while r*Pp*Rcys1_RMRK lacks this capability.

### 3.7. Comparison of the Effects of rPpRcys1_RMRK and rPpRcys1 on Bacterial Morphology

**Figure 7:**
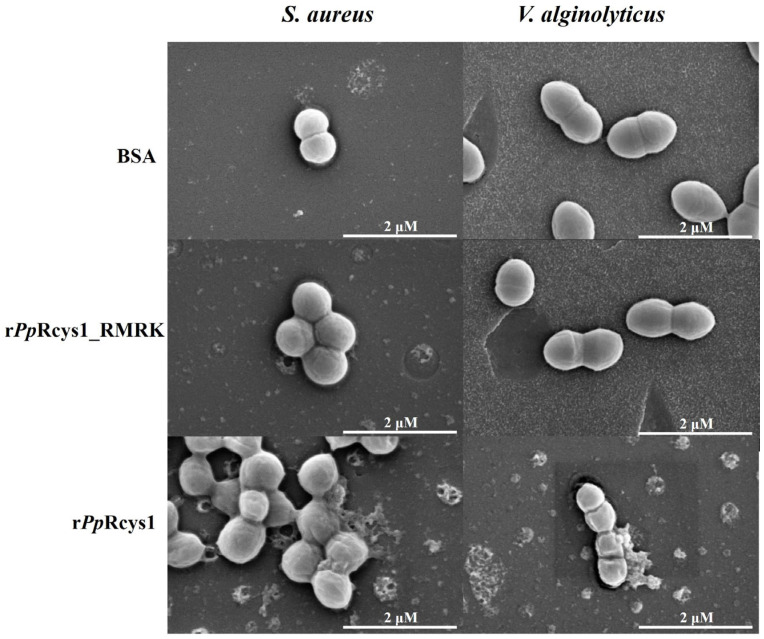
Observation of morphological alterations in bacterial cells following treatment with r*Pp*Rcys1 and r*Pp*Rcys1_RMRK. Bacteria at a concentration of approximately 10^6^ CFU/mL were exposed to 64 μM of r*Pp*Rcys1 or r*Pp*Rcys1_RMRK for 2 h and examined using scanning electron microscopy (SEM). BSA served as the control, and scale bars represent 2 μm.

Scanning electron microscopy revealed that bacteria in the control group and those treated with 64 μM r*Pp*Rcys1_RMRK had regular morphologies. The bacteria treated with r*Pp*Rcys1 became irregular in shape and showed a significant increase in wrinkles (Figure 7).

## 4. Discussion

The activity and selectivity of AMPs are strongly influenced by their primary structure, spatial conformation, and physicochemical properties. A systematic analysis of the relationship between these structural features and biological functions can not only clarify the mechanisms underlying antimicrobial activity but also provide essential guidance for the rational design of novel AMPs with high efficacy and low toxicity [50,51]. Molecular dynamics simulations offer a powerful approach to visualize, at the atomic level, the dynamic interactions between AMPs and their targets, including conformational changes, modes of membrane disruption, and the functional contributions of key residues [23,25]. This approach overcomes the spatial and temporal limitations of experimental techniques, thus enabling a deeper understanding of the structure–activity relationships of AMPs [52,53].

The result of MMPBSA revealed that four key positively charged residues (ARG40, LYS55, LYS90, and LYS93) are critical for the antibacterial activity of the antimicrobial peptide *Pp*Rcys1 (Figure 1A). Our previous research findings indicate that the ARG40-LYS55 region of *Pp*Rcys1 constitutes a critical segment underlying its membrane penetration capability [28]. Furthermore, conformational analysis derived from molecular dynamics simulations demonstrates that LYS90 and LYS93 are capable of anchoring within the cell membrane [28], a result consistent with the MMPBSA computational data. In *Pp*Rcys1, arginine and lysine are basic amino acids that carry a positive charge under physiological conditions [54]. Serine was selected as the substitution target due to its polar, uncharged nature [55]. This substitution effectively eliminates the positive charges from the side chains while minimally affecting the size and polarity of the residues, thereby enabling a specific assessment of the contribution of the positive charge. Although alanine is commonly used in site-directed mutagenesis, it is hydrophobic [29,56], whereas lysine, arginine, and serine are all hydrophilic amino acids [57,58]. Serine represents a more appropriate substitute to maintain hydrophilicity while removing charge. Mutation of these residues to serine resulted in the mutant *Pp*Rcys1_RMRK, which completely lost its antibacterial activity and exhibited markedly reduced interaction with bacterial membranes and diminished membrane-disrupting ability. These findings suggest that identifying key functional residues of AMPs through MD simulations combined with free energy calculations is an effective strategy.

Among the commonly employed methods for optimizing antibody affinity and peptide activity, single-point missense mutagenesis represents a widely adopted strategy [59,60]. However, existing studies have demonstrated only a weak correlation between predicted outcomes of single-point missense mutations and experimentally measured changes in biological activity [61,62]. Consequently, in recent years, increasing attention has been directed toward multi-site mutagenesis approaches aimed at substantially improving the functional performance of antibodies and enzymes. For example, Huang et al. integrated via FuncLib (for mutant enzyme design) and Rosetta Cartesian_ddg (for free energy calculation) to develop a novel strategy for multi-site mutagenesis within the binding pocket region, thereby enhancing both the reaction rate and yield in lipase-catalyzed biodiesel production [62]. Additionally, mmCSM-AB leverages graph-based representations incorporating sequence, structural, and physicochemical features to rapidly and comprehensively assess the effects of multiple mutation combinations in antibody–antigen complexes [61]. Notably, the present study does not aim to enhance the activity of *Pp*Rcys1. Given that the mature form of *Pp*Rcys1 comprises 85 amino acids, its molecular weight exceeds that of melittin and LL37 [63,64]. Molecular dynamics simulations reveal that its translocation into the lipid membrane markedly slows after 300 ns [28]. Thus, the primary objective of this work is to identify key functional regions within *Pp*Rcys1. MMPBSA analysis identified ARG40, LYS55, LYS90, and LYS93 as residues playing significant roles in membrane insertion and interaction with membrane components (Figure 1A). Accordingly, these four residues were simultaneously mutated. The results showed a marked reduction in the antibacterial activity of *Pp*Rcys1 upon quadruple mutation. Subsequently, we plan to further investigate the functional contribution of the ARG40–LYS93 region and examine the synergistic effects among cationic amino acids in *Pp*Rcys1 through systematic single-point mutagenesis experiments.

AMP activity is closely associated with their physicochemical properties [65,66]. Compared with *Pp*Rcys1, the antibacterial activity of *Pp*Rcys1_RMRK was significantly reduced. Our previous study demonstrated that *Pp*Rcys1 kills bacteria primarily by disrupting cell membranes [28]. The initial electrostatic interaction between AMPs and the negatively charged bacterial membrane is typically the first step in AMP-mediated membrane targeting [65,67]. In *Pp*Rcys1, mutation of ARG40, LYS55, LYS90, and LYS93 to neutral residues substantially decreased the overall charge of the peptide, thus weakening the initial electrostatic attraction between the peptide and the negatively charged bacterial membrane. Hydrophobicity is a key factor influencing AMP activity [68,69]. For example, adding Trp-Trp-Trp tags to the N- or C-terminus of GKH17 enhanced the peptide’s hydrophobicity, significantly reducing its MIC against *S. aureus* [68,70]. In contrast, mutation of ARG40, LYS55, LYS90, and LYS93 to serine in *Pp*Rcys1 substantially decreased its hydrophobicity. This likely reduced the ability of the peptide segments to penetrate the membrane environment and insert into the lipid bilayer [71,72]. Western blot and ELISA experiments further confirmed that the mutant’s binding to bacteria and membrane mimics was significantly reduced (Figure 4), corroborating the predictions from the MD simulations at the biochemical level. Together, these results show that ARG40, LYS55, LYS90, and LYS93 are key residues mediating membrane interactions in *Pp*Rcys1.

Based on the MD simulation results, we hypothesized that mutation of ARG40, LYS55, LYS90, and LYS93 to serine would prevent *Pp*Rcys1 from binding to bacterial membranes, thus abolishing its membrane-disrupting activity. To test this hypothesis, we performed experimental validations, including membrane permeability assays, membrane depolarization experiments, and bacterial morphology observations [73,74,75]. *Pp*Rcys1 induced membrane leakage, depolarization of bacterial cell membranes, and surface shrinkage of bacterial cells, whereas *Pp*Rcys1_RMRK did not. These findings confirmed that ARG40, LYS55, LYS90, and LYS93 are critical residues for *Pp*Rcys1’s membrane-binding and membrane-disrupting activities.

LC–MS is a commonly used method for identifying recombinant-expressed proteins, typically requiring enzymatic cleavage into smaller peptide fragments prior to detection [76]. In this study, trypsin was used, which specifically cleaves at the C-terminal of arginine and lysine residues [14,77]. After trypsin treatment, wild-type *Pp*Rcys1 was cleaved at the C-termini of ARG40, ARG59, and LYS93, generating three peptide fragments. In *Pp*Rcys1_RMRK, ARG40 and LYS93 were mutated to serine, preventing these sites from being recognized and cleaved by trypsin. Consequently, under identical recombinant expression and purification conditions, only one peptide fragment could be detected in the mutant.

Building on previous results, this study further explored the structure–activity relationship of *Pp*Rcys1, identified its functional core, and provided precise targets for subsequent rational, structure-based design. Rather than mutating these key residues, future strategies could focus on protecting them from protease hydrolysis. The integration of MD simulations with experimental biology could guide AMP optimization [23]. Although mutations lead to inactivation, they emphasize the importance of these residues and provide a scientific basis for the deletion of redundant parts of macromolecular antimicrobial peptides. Future approaches, such as incorporation of D-amino acids or peptide cyclization, could preserve these key residues while enhancing overall activity and reducing biological toxicity of the peptide.

## 5. Conclusions

This study confirmed that ARG40, LYS55, LYS90, and LYS93 are essential residues for *Pp*Rcys1’s antibacterial activity, primarily by mediating the initial binding and interaction between the peptide and the bacterial cell membrane. For future efforts to optimize activity and reduce biological toxicity, these residues should be preserved and strategically modified to prevent trypsin cleavage.

## Figures and Tables

**Table 1:** Physicochemical properties of *Pp*Rcys1_RMRK and *Pp*Rcys1.

Peptides	Molecular Weight (kDa)	Protein Isoelectric Point	Net Charge	Grand Average Hydropathy	Wimley–White Whole-Residue Hydrophobicity	Boman Index
*Pp*Rcys1	11.18	8.50	+4.5	0.48	3.03	0.32
*Pp*Rcys1_RMRK	10.98	6.65	+0.5	0.60	−0.23	0.15

**Table 2:** Minimal inhibitory concentrations (MICs) of r*Pp*Rcys1 and *rPpRcys1_RMRK* against Gram-positive and Gram-negative bacteria.

Microorganism		Minimal Inhibitory Concentrations (μM)		
		r*Pp*Rcys1	r*Pp*Rcys1_RMRK	Ampicillin
Gram-positive bacteria	*S. aureus*	8	-	2
	*Bacillus* sp. T2	8	-	
	*S. agalactiae*	16	-	4
Gram-negative bacteria ^−^	*A. hydrophila*	32	-	128
	*Acinetobacter* sp. L32	32	-	-
	*E. coli*	16	-	64
	*V. alginolyticus*	16	-	-

“−” denotes the absence of inhibitory activity for the compound at a concentration of 64 μM.

## Data Availability

All data generated or analyzed during this study are included in this published article, and further inquiries can be directed to the corresponding authors.

## Supplementary Materials

The following supporting information can be downloaded at: https://www.mdpi.com/article/10.3390/biom15111617/s1, Figure S1: pSmartI-*Pp*Rcys1_RMRK map; Figure S2: The pLDDT analysis of PpRcys1 and PpRcys1_RMRK were predicted using AlphaFold2. Figure S3: Representative snapshots from the MD simulation of PpRcys1 in aqueous solution. Figure S4: The electrostatic and van der Waals interactions between *Pp*Rcys1 and the cell membrane (A), along with the convergence analysis (B). Figure S5: Original WB figure of Figure 4A; Table S1: General Primers of pSmartI; Table S2: PCR amplification program; Table S3: Encoding sequences of the mature peptides in *Pp*Rcys1 and *Pp*Rcys1_RMRK were optimized based on the codon preference of *E. coli*.

## References

[1] Manage P.M. (2018). Heavy Use of Antibiotics in Aquaculture: Emerging Human and Animal Health Problems—A Review. Sri Lanka J. Aquat. Sci..

[2] Rigos G., Kogiannou D., Knowles M.E., Anelich L.E., Boobis A.R., Popping B. (2023). Chapter 9—Antimicrobial Drugs in Aquaculture: Use and Abuse. Present Knowledge in Food Safety.

[3] Chen P., Ye T., Li C., Praveen P., Hu Z., Li W., Shang C. (2024). Embracing the Era of Antimicrobial Peptides with Marine Organisms. Nat. Prod. Rep..

[4] Li C., Warren R.L., Birol I. (2023). Models and Data of AMPlify: A Deep Learning Tool for Antimicrobial Peptide Prediction. BMC Res. Notes.

[5] Wang G., Li X., Wang Z. (2016). APD3: The Antimicrobial Peptide Database as a Tool for Research and Education. Nucleic Acids Res..

[6] Oliveira Júnior N.G., Souza C.M., Buccini D.F., Cardoso M.H., Franco O.L. (2025). Antimicrobial Peptides: Structure, Functions and Translational Applications. Nat. Rev. Microbiol..

[7] Zou F., Tan C., Shinali T.S., Zhang B., Zhang L., Han Z., Shang N. (2023). Plant Antimicrobial Peptides: A Comprehensive Review of Their Classification, Production, Mode of Action, Functions, Applications, and Challenges. Food Funct..

[8] Barreto-Santamaría A., Patarroyo M.E., Curtidor H. (2019). Designing and Optimizing New Antimicrobial Peptides: All Targets Are Not the Same. Crit. Rev. Clin. Lab. Sci..

[9] Tan P., Lai Z., Zhu Y., Shao C., Akhtar M.U., Li W., Zheng X., Shan A. (2020). Multiple Strategy Optimization of Specifically Targeted Antimicrobial Peptide Based on Structure–Activity Relationships to Enhance Bactericidal Efficiency. ACS Biomater. Sci. Eng..

[10] Juba M.L., Porter D.K., Williams E.H., Rodriguez C.A., Barksdale S.M., Bishop B.M. (2015). Helical Cationic Antimicrobial Peptide Length and Its Impact on Membrane Disruption. Biochim. Biophys. Acta (BBA)-Biomembr..

[11] Hall K., Lee T., Aguilar M. (2011). The Role of Electrostatic Interactions in the Membrane Binding of Melittin. J. Mol. Recognit..

[12] He S., Deber C.M. (2024). Interaction of Designed Cationic Antimicrobial Peptides with the Outer Membrane of Gram-Negative Bacteria. Sci. Rep..

[13] Felsztyna I., Galassi V.V., Wilke N. (2025). Selectivity of Membrane-Active Peptides: The Role of Electrostatics and Other Membrane Biophysical Properties. Biophys. Rev..

[14] Olsen J.V., Ong S.-E., Mann M. (2004). Trypsin Cleaves Exclusively C-Terminal to Arginine and Lysine Residues. Mol. Cell. Proteom..

[15] Jiang Z., Kullberg B.J., Van Der Lee H., Vasil A.I., Hale J.D., Mant C.T., Hancock R.E.W., Vasil M.L., Netea M.G., Hodges R.S. (2008). Effects of Hydrophobicity on the Antifungal Activity of A-helical Antimicrobial Peptides. Chem. Biol. Drug Des..

[16] He S., Stone T.A., Deber C.M. (2021). Uncoupling Amphipathicity and Hydrophobicity: Role of Charge Clustering in Membrane Interactions of Cationic Antimicrobial Peptides. Biochemistry.

[17] Yin L.M., Edwards M.A., Li J., Yip C.M., Deber C.M. (2012). Roles of Hydrophobicity and Charge Distribution of Cationic Antimicrobial Peptides in Peptide-Membrane Interactions. J. Biol. Chem..

[18] Ruiz J., Calderon J., Rondón-Villarreal P., Torres R. (2014). Analysis of Structure and Hemolytic Activity Relationships of Antimicrobial Peptides (AMPs). Advances in Computational Biology, Proceedings of the 2nd Colombian Congress on Computational Biology and Bioinformatics (CCBCOL), Manizales, Colombia, 25–27 September 2013.

[19] Ciulla M.G., Gelain F. (2023). Structure–Activity Relationships of Antibacterial Peptides. Microb. Biotechnol..

[20] Ahn H., Cho W., Kang S.-H., Ko S.-S., Park M.-S., Cho H., Lee K.-H. (2006). Design and Synthesis of Novel Antimicrobial Peptides on the Basis of α Helical Domain of Tenecin 1, an Insect Defensin Protein, and Structure–Activity Relationship Study. Peptides.

[21] Wang Y., Zhao T., Wei D., Strandberg E., Ulrich A.S., Ulmschneider J.P. (2014). How Reliable Are Molecular Dynamics Simulations of Membrane Active Antimicrobial Peptides?. Biochim. Biophys. Acta (BBA)-Biomembr..

[22] Ulmschneider J.P., Ulmschneider M.B. (2018). Molecular Dynamics Simulations Are Redefining Our View of Peptides Interacting with Biological Membranes. Acc. Chem. Res..

[23] Yuan H., Lyu Y., Cui X., Zhang C., Meng Q. (2024). How Antimicrobial Peptide Indolicidin and Its Derivatives Interact with Phospholipid Membranes: Molecular Dynamics Simulation. J. Mol. Struct..

[24] Nosé S. (1984). A Molecular Dynamics Method for Simulations in the Canonical Ensemble. Mol. Phys..

[25] Cao Q., Ge C., Wang X., Harvey P.J., Zhang Z., Ma Y., Wang X., Jia X., Mobli M., Craik D.J. (2023). Designing Antimicrobial Peptides Using Deep Learning and Molecular Dynamic Simulations. Brief. Bioinform..

[26] Sun D., Peyear T.A., Bennett W.F.D., Andersen O.S., Lightstone F.C., Ingólfsson H.I. (2019). Molecular Mechanism for Gramicidin Dimerization and Dissociation in Bilayers of Different Thickness. Biophys. J..

[27] Chen R., Mark A.E. (2011). The Effect of Membrane Curvature on the Conformation of Antimicrobial Peptides: Implications for Binding and the Mechanism of Action. Eur. Biophys. J..

[28] He Z., Fei Z., Shi H., Huang M., Wei L., Wang J., He P., Zhang W. (2025). Heterologous Expression and Antimicrobial Mechanism of a Cysteine-Rich Peptide from Barnacle Pollicipes Pollicipes. Microorganisms.

[29] Wan H., Li Y., Fan Y., Meng F., Chen C., Zhou Q. (2012). A Site-Directed Mutagenesis Method Particularly Useful for Creating Otherwise Difficult-to-Make Mutants and Alanine Scanning. Anal. Biochem..

[30] Etayash H., Azmi S., Dangeti R., Kaur K. (2016). Peptide Bacteriocins-Structure Activity Relationships. Curr. Top. Med. Chem..

[31] Avitabile C., Netti F., Orefice G., Palmieri M., Nocerino N., Malgieri G., D’Andrea L.D., Capparelli R., Fattorusso R., Romanelli A. (2013). Design, Structural and Functional Characterization of a Temporin-1b Analog Active against Gram-Negative Bacteria. Biochim. Biophys. Acta (BBA)-Gen. Subj..

[32] Zhang W., Wei L., Chen P., Ning B., Wang J., He P., Shang C., Yu D. (2024). Discovery and Characterization of an Atypical Crustin Antimicrobial Peptide from Pollicipes Pollicipes. Mar. Drugs.

[33] Best R.B., Zhu X., Shim J., Lopes P.E.M., Mittal J., Feig M., MacKerell Jr A.D. (2012). Optimization of the Additive CHARMM All-Atom Protein Force Field Targeting Improved Sampling of the Backbone ϕ, ψ and Side-Chain Χ1 and Χ2 Dihedral Angles. J. Chem. Theory Comput..

[34] Murzyn K., Róg T., Pasenkiewicz-Gierula M. (2005). Phosphatidylethanolamine-Phosphatidylglycerol Bilayer as a Model of the Inner Bacterial Membrane. Biophys. J..

[35] Balatti G.E., Martini M.F., Pickholz M. (2018). A Coarse-Grained Approach to Studying the Interactions of the Antimicrobial Peptides Aurein 1.2 and Maculatin 1.1 with POPG/POPE Lipid Mixtures. J. Mol. Model..

[36] Berendsen H.J.C., Postma J.P.M., van Gunsteren W.F., DiNola A., Haak J.R. (1984). Molecular Dynamics with Coupling to an External Bath. J. Chem. Phys..

[37] Abraham M.J., Murtola T., Schulz R., Páll S., Smith J.C., Hess B., Lindahl E. (2015). GROMACS: High Performance Molecular Simulations through Multi-Level Parallelism from Laptops to Supercomputers. SoftwareX.

[38] Humphrey W., Dalke A., Schulten K. (1996). VMD: Visual Molecular Dynamics. J. Mol. Graph..

[39] Bussi G., Donadio D., Parrinello M. (2007). Canonical Sampling through Velocity Rescaling. J. Chem. Phys..

[40] Bernetti M., Bussi G. (2020). Pressure Control Using Stochastic Cell Rescaling. J. Chem. Phys..

[41] Essmann U., Perera L., Berkowitz M.L., Darden T., Lee H., Pedersen L.G. (1995). A Smooth Particle Mesh Ewald Method. J. Chem. Phys..

[42] Homeyer N., Gohlke H. (2012). Free Energy Calculations by the Molecular Mechanics Poisson− Boltzmann Surface Area Method. Mol. Inf..

[43] Cramer P. (2021). AlphaFold2 and the Future of Structural Biology. Nat. Struct. Mol. Biol..

[44] Cornett J.B., Shockman G.D. (1978). Cellular Lysis of Streptococcus Faecalis Induced with Triton X-100. J. Bacteriol..

[45] Sung K., Khan S.A., Nawaz M.S., Khan A.A. (2003). A Simple and Efficient Triton X-100 Boiling and Chloroform Extraction Method of RNA Isolation from Gram-Positive and Gram-Negative Bacteria. FEMS Microbiol. Lett..

[46] Te Winkel J.D., Gray D.A., Seistrup K.H., Hamoen L.W., Strahl H. (2016). Analysis of Antimicrobial-Triggered Membrane Depolarization Using Voltage Sensitive Dyes. Front. Cell Dev. Biol..

[47] Epand R.F., Pollard J.E., Wright J.O., Savage P.B., Epand R.M. (2010). Depolarization, Bacterial Membrane Composition, and the Antimicrobial Action of Ceragenins. Antimicrob. Agents Chemother..

[48] Yeaman M.R., Yount N.Y. (2003). Mechanisms of Antimicrobial Peptide Action and Resistance. Pharmacol. Rev..

[49] Chen Y., Yi M., Wang Y., Yao L., Ji G., Gao Z. (2025). Identification of a Novel Antimicrobial Peptide from Amphioxus Ribosomal Protein L27. Fish. Shellfish Immunol..

[50] Li Y., Yu J. (2015). Research Progress in Structure-Activity Relationship of Bioactive Peptides. J. Med. Food.

[51] Bakare O.O., Gokul A., Fadaka A.O., Wu R., Niekerk L.-A., Barker A.M., Keyster M., Klein A. (2022). Plant Antimicrobial Peptides (PAMPs): Features, Applications, Production, Expression, and Challenges. Molecules.

[52] Karagöl A., Karagöl T., Smorodina E., Zhang S. (2024). Structural Bioinformatics Studies of Glutamate Transporters and Their AlphaFold2 Predicted Water-Soluble QTY Variants and Uncovering the Natural Mutations of L-> Q, I-> T, F-> Y and Q-> L, T-> I and Y-> F. PLoS ONE.

[53] Buel G.R., Walters K.J. (2022). Can AlphaFold2 Predict the Impact of Missense Mutations on Structure?. Nat. Struct. Mol. Biol..

[54] Li L., Vorobyov I., Allen T.W. (2013). The Different Interactions of Lysine and Arginine Side Chains with Lipid Membranes. J. Phys. Chem. B.

[55] Gisdon F.J., Bombarda E., Ullmann G.M. (2022). Serine and Cysteine Peptidases: So Similar, yet Different. How the Active-Site Electrostatics Facilitates Different Reaction Mechanisms. J. Phys. Chem. B.

[56] Moreira I.S., Fernandes P.A., Ramos M.J. (2007). Computational Alanine Scanning Mutagenesis—An Improved Methodological Approach. J. Comput. Chem..

[57] Trevino S.R., Scholtz J.M., Pace C.N. (2007). Amino Acid Contribution to Protein Solubility: Asp, Glu, and Ser Contribute More Favorably than the Other Hydrophilic Amino Acids in RNase Sa. J. Mol. Biol..

[58] Mitaku S., Hirokawa T., Tsuji T. (2002). Amphiphilicity Index of Polar Amino Acids as an Aid in the Characterization of Amino Acid Preference at Membrane–Water Interfaces. Bioinformatics.

[59] Conti E., Kuriyan J. (2000). Crystallographic Analysis of the Specific yet Versatile Recognition of Distinct Nuclear Localization Signals by Karyopherin α. Structure.

[60] Sulea T., Hussack G., Ryan S., Tanha J., Purisima E.O. (2018). Application of Assisted Design of Antibody and Protein Therapeutics (ADAPT) Improves Efficacy of a Clostridium Difficile Toxin A Single-Domain Antibody. Sci. Rep..

[61] Myung Y., Pires D.E.V., Ascher D.B. (2020). MmCSM-AB: Guiding Rational Antibody Engineering through Multiple Point Mutations. Nucleic Acids Res..

[62] Huang J., Xie X., Zheng W., Xu L., Yan J., Wu Y., Yang M., Yan Y. (2024). In Silico Design of Multipoint Mutants for Enhanced Performance of Thermomyces Lanuginosus Lipase for Efficient Biodiesel Production. Biotechnol. Biofuels Bioprod..

[63] Dempsey C.E. (1990). The Actions of Melittin on Membranes. Biochim. Biophys. Acta (BBA)-Rev. Biomembr..

[64] Elssner A., Duncan M., Gavrilin M., Wewers M.D. (2004). A Novel P2X7 Receptor Activator, the Human Cathelicidin-Derived Peptide LL37, Induces IL-1β Processing and Release. J. Immunol..

[65] Torrent M., Andreu D., Nogués V.M., Boix E. (2011). Connecting Peptide Physicochemical and Antimicrobial Properties by a Rational Prediction Model. PLoS ONE.

[66] Giangaspero A., Sandri L., Tossi A. (2001). Amphipathic α Helical Antimicrobial Peptides. A Systematic Study of the Effects of Structural and Physical Properties on Biological Activity. Eur. J. Biochem..

[67] Travkova O.G., Moehwald H., Brezesinski G. (2017). The Interaction of Antimicrobial Peptides with Membranes. Adv. Colloid Interface Sci..

[68] Schmidtchen A., Pasupuleti M., Malmsten M. (2014). Effect of Hydrophobic Modifications in Antimicrobial Peptides. Adv. Colloid Interface Sci..

[69] Gagat P., Ostrówka M., Duda-Madej A., Mackiewicz P. (2024). Enhancing Antimicrobial Peptide Activity through Modifications of Charge, Hydrophobicity, and Structure. Int. J. Mol. Sci..

[70] Schmidtchen A., Pasupuleti M., Mörgelin M., Davoudi M., Alenfall J., Chalupka A., Malmsten M. (2009). Boosting Antimicrobial Peptides by Hydrophobic Oligopeptide End Tags. J. Biol. Chem..

[71] Jindal M.H., Le C.F., Mohd Yusof M.Y., Sekaran S.D. (2014). Net Charge, Hydrophobicity and Specific Amino Acids Contribute to the Activity of Antimicrobial Peptides. J. Health Transl. Med..

[72] Pirtskhalava M., Vishnepolsky B., Grigolava M., Managadze G. (2021). Physicochemical Features and Peculiarities of Interaction of AMP with the Membrane. Pharmaceuticals.

[73] Espeche J.C., Varas R., Maturana P., Cutro A.C., Maffía P.C., Hollmann A. (2024). Membrane Permeability and Antimicrobial Peptides: Much More than Just Making a Hole. Pept. Sci..

[74] Penyige A., Matkó J., Deák E., Bodnár A., Barabás G. (2002). Depolarization of the Membrane Potential by β-Lactams as a Signal to Induce Autolysis. Biochem. Biophys. Res. Commun..

[75] Zhang W., Xu X., Zhang J., Ye T., Zhou Q., Xu Y., Li W., Hu Z., Shang C. (2022). Discovery and Characterization of a New Crustin Antimicrobial Peptide from Amphibalanus Amphitrite. Pharmaceutics.

[76] Lubec G., Afjehi-Sadat L. (2007). Limitations and Pitfalls in Protein Identification by Mass Spectrometry. Chem. Rev..

[77] Zhao X., Zhang M., Muhammad I., Cui Q., Zhang H., Jia Y., Xu Q., Kong L., Ma H. (2021). An Antibacterial Peptide with High Resistance to Trypsin Obtained by Substituting D-Amino Acids for Trypsin Cleavage Sites. Antibiotics.

