# Supplementary material for: Screening and Validation of Functional Residues of the Antimicrobial Peptide PpRcys1
Source: Biomolecules. 2025 Nov 18;15(11):1617. doi: 10.3390/biom15111617 (PMC12650370; doi:10.3390/biom15111617)
Supplement: Supplementary file 1 [file biomolecules-15-01617-s001.zip › biomolecules-3957884 - WB Original Images/Origin WB image/Illustration of the original image.pdf]

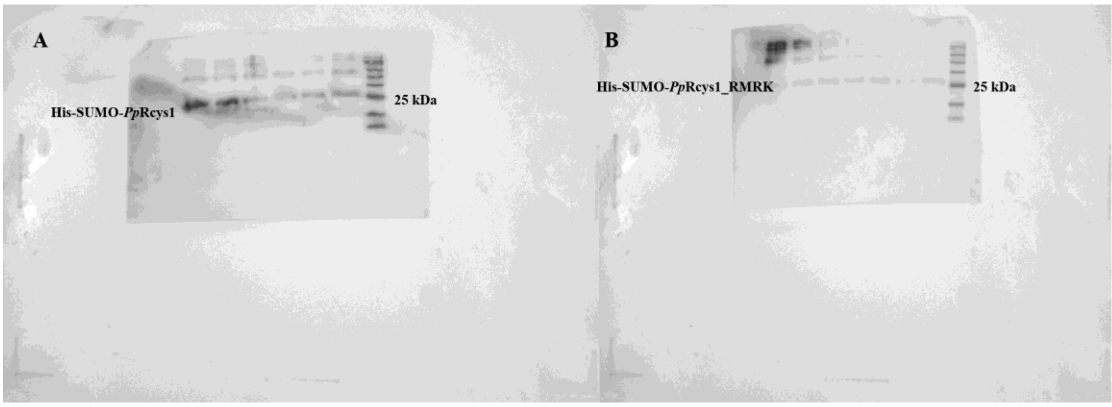

Illustration of the original image, Original WB figure of Figure 4A. (A) original WB images of Figure 4A upper, His-SUMO-*Pp*Rcys1 were detected by western blotting after treatment with *S. aureus*. (B) original WB images of Figure 4A lower, His-SUMO-*Pp*Rcys1_RMRK were detected by western blotting after treatment with *S. aureus.*
