# Supplementary material for: Screening and Validation of Functional Residues of the Antimicrobial Peptide PpRcys1
Source: Biomolecules. 2025 Nov 18;15(11):1617. doi: 10.3390/biom15111617 (PMC12650370; doi:10.3390/biom15111617)
Supplement: Supplementary file 1 [file biomolecules-15-01617-s001.zip › biomolecules-3957884-supplementary/supplementary.pdf]

Table S1. General Primers of pSmartI

Primers	5'-3'
EF	TTA AGA TTC TTG TAC GAC GG
ER	TGC TAG TTA TTG CTC AGC GG

Table S2. PCR amplification program

Program	Temperature	Time	Cycles
Initial denaturation	95 ℃	5 min	1
Denaturation	95 ℃	30 s	35
Anneal	51 ℃	30 s	
Extend	72 ℃	1 min	
Final extension	72 ℃	5 min	1

Table S3. Encoding sequences of the mature peptides in *Pp*Rcys1 and *Pp*Rcys1_RMRK were optimized based on the codon preference of *E. coli*.

Sequences name	Encoding
*Pp*Rcys1	GGATCCCAGACCTGTCTGAATCGCCCGGGTCAG<br>TGCCCGACCTTTATTAGCCCGTTTAGTCTGCCGC<br>GCACCCTGTGTAGCACCGATTGCGATTGCAATC<br>TGAGCCATCATAAAGGCACCTGGCGTTGTTGTC<br>CGACCTTTGTTGGTGACGTGTGTCTGCCGCCGT<br>GCAATCCGGTTTGCCCGCTGTTTAGCGTTTGTAC<br>CCTGGTGAGTCAGGTGAAACCGTGGAAAAGCT<br>ATTGCGTGTTTGCCGGCCCGACCAAAAGTTAAC<br>TCGAG
*Pp*Rcys1_RMRK	GGATCCCAGACCTGTCTGAATCGCCCGGGTCAG<br>TGTCCGACCTTTATTAGCCCGTTTAGCCTGCCGA<br>GTACCCTGTGCAGCACCGATTGTGATTGCAATC<br>TGAGTCATCATAGCGGTACATGGCGTTGCTGTC<br>CGACCTTCGTGGGTGACGTGTGCCTGCCGCCGT<br>GCAATCCGGTGTGCCCGCTGTTTAGTGTGTGTA<br>CCCTGGTTAGCCAGGTTAGTCCGTGGAGCAGTT<br>ATTGTGTTTTCGCAGGCCCGACCAAAAGTTAAC<br>TCGAG

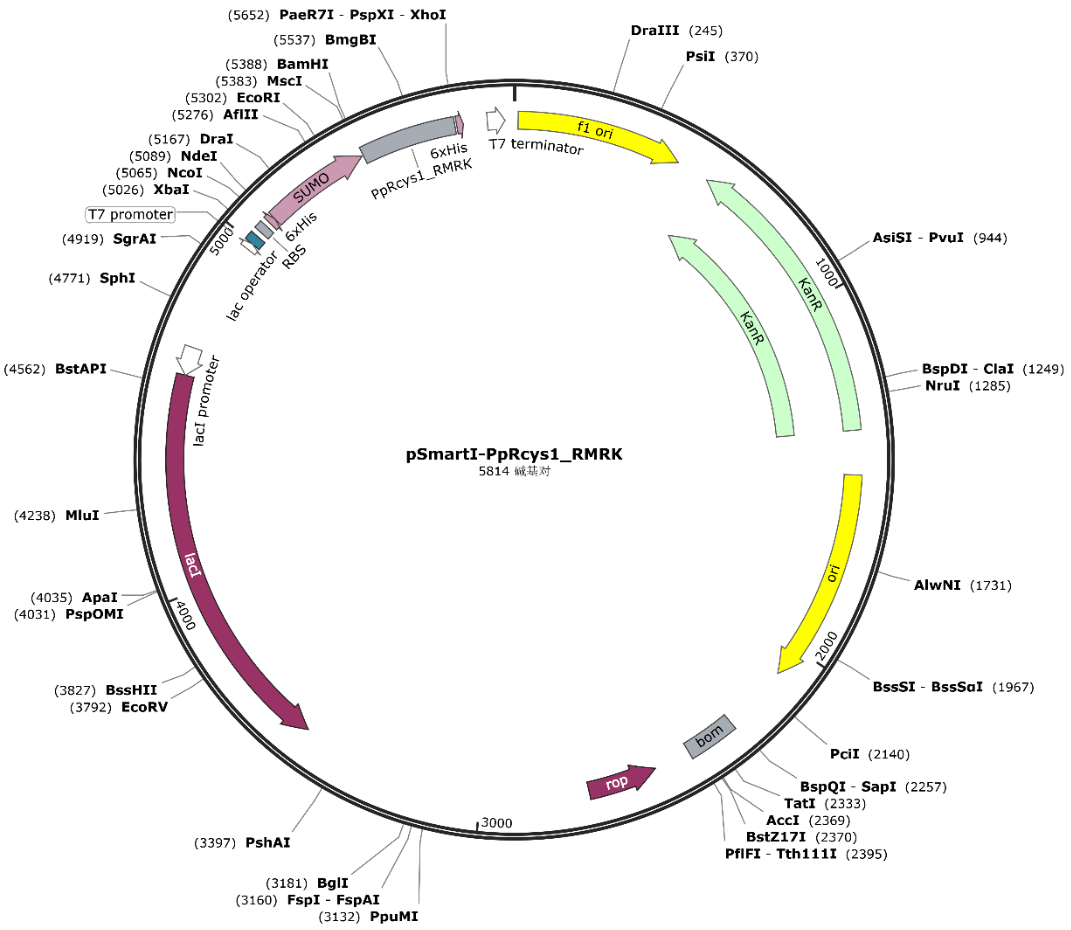

Supplementary Figure S1. pSmartI-*Pp*Rcys1_RMRK map

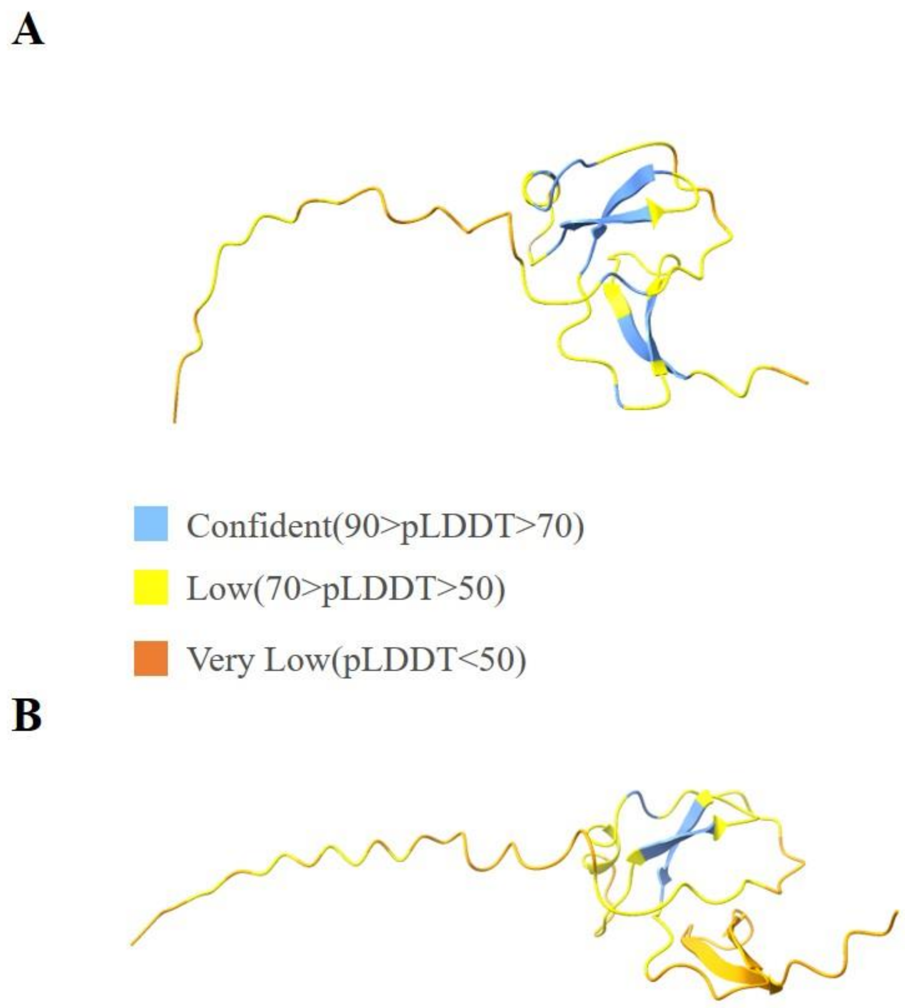

Supplementary Figure S2. The pLDDT analysis of *Pp*Rcys1 and *Pp*Rcys1_RMRK were predicted using AlphaFold2.

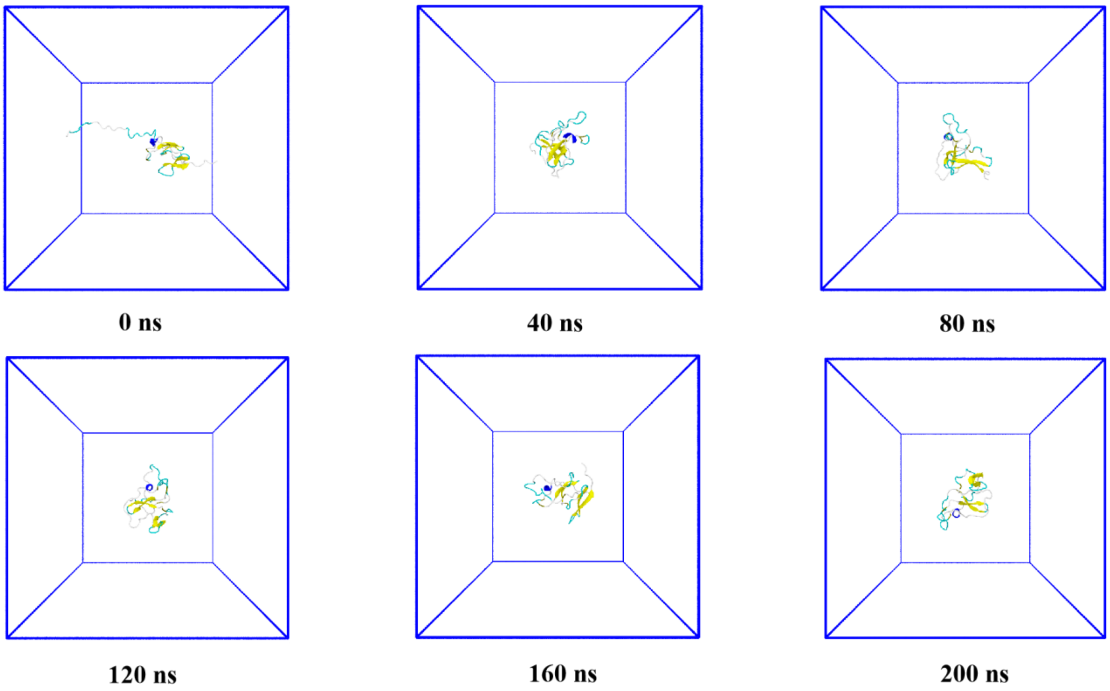

Supplementary Figure S3. Representative snapshots from the MD simulation of *Pp*Rcys1 in aqueous solution

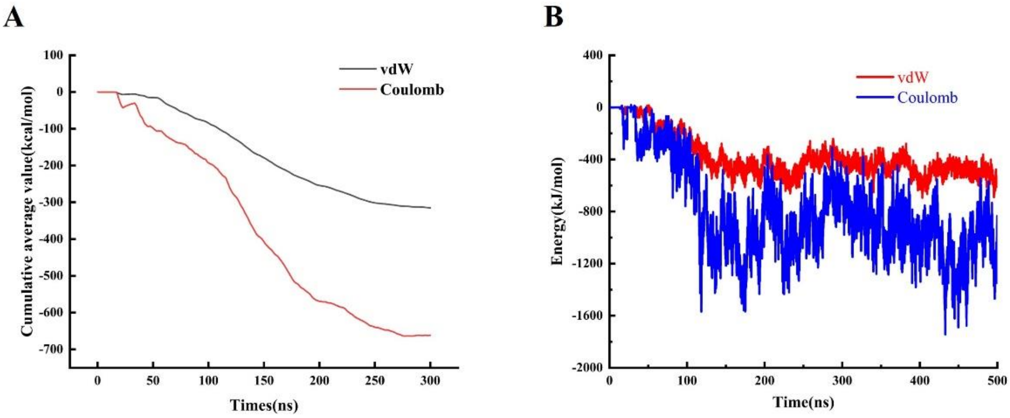

Supplementary Figure S4: The electrostatic and van der Waals interactions between PpRcys1 and the cell membrane (A), along with the convergence analysis (B).

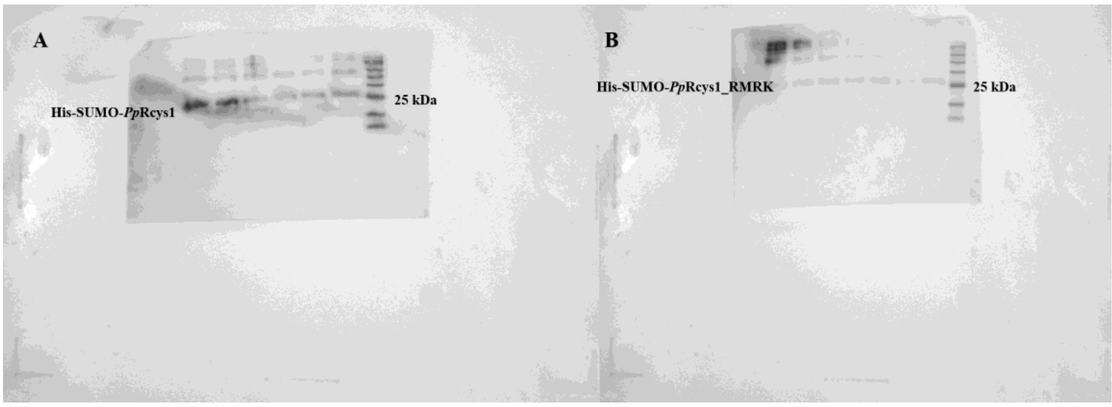

Supplementary Figure S5. Original WB figure of Figure 4A. (A) His-SUMO-*Pp*Rcys1 were detected by western blotting after treatment with *S. aureus*. (B) His-SUMO-*Pp*Rcys1_RMRK were detected by western blotting after treatment with *S. aureus.*
