# Supplementary figures and images for: Screening and Validation of Functional Residues of the Antimicrobial Peptide PpRcys1
Source: Biomolecules. 2025 Nov 18;15(11):1617. doi: 10.3390/biom15111617 (PMC12650370; doi:10.3390/biom15111617)

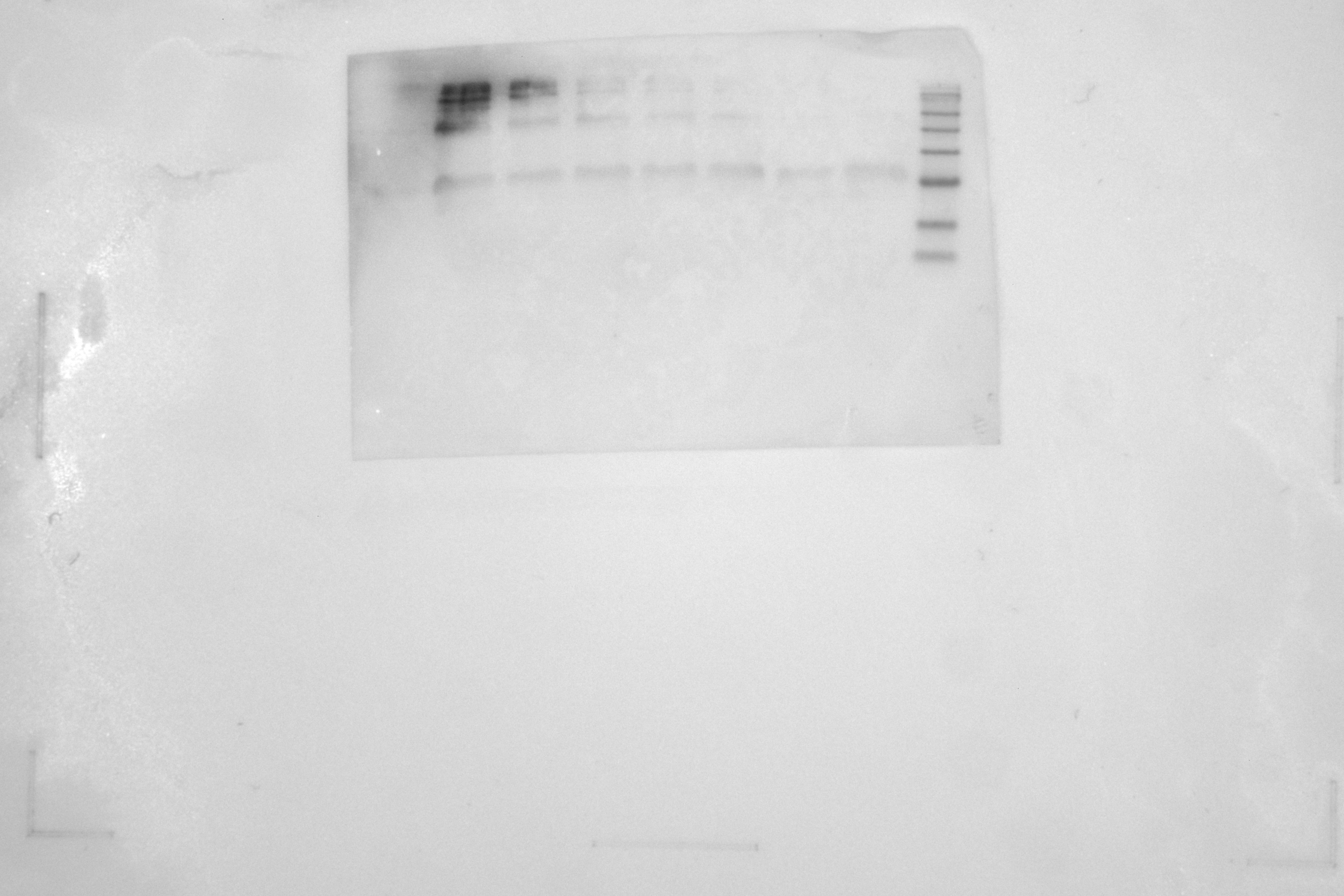

Supplement: Supplementary file 1 [file biomolecules-15-01617-s001.zip › biomolecules-3957884 - WB Original Images/Origin WB image/original WB images of Figure 4A lower.tif]

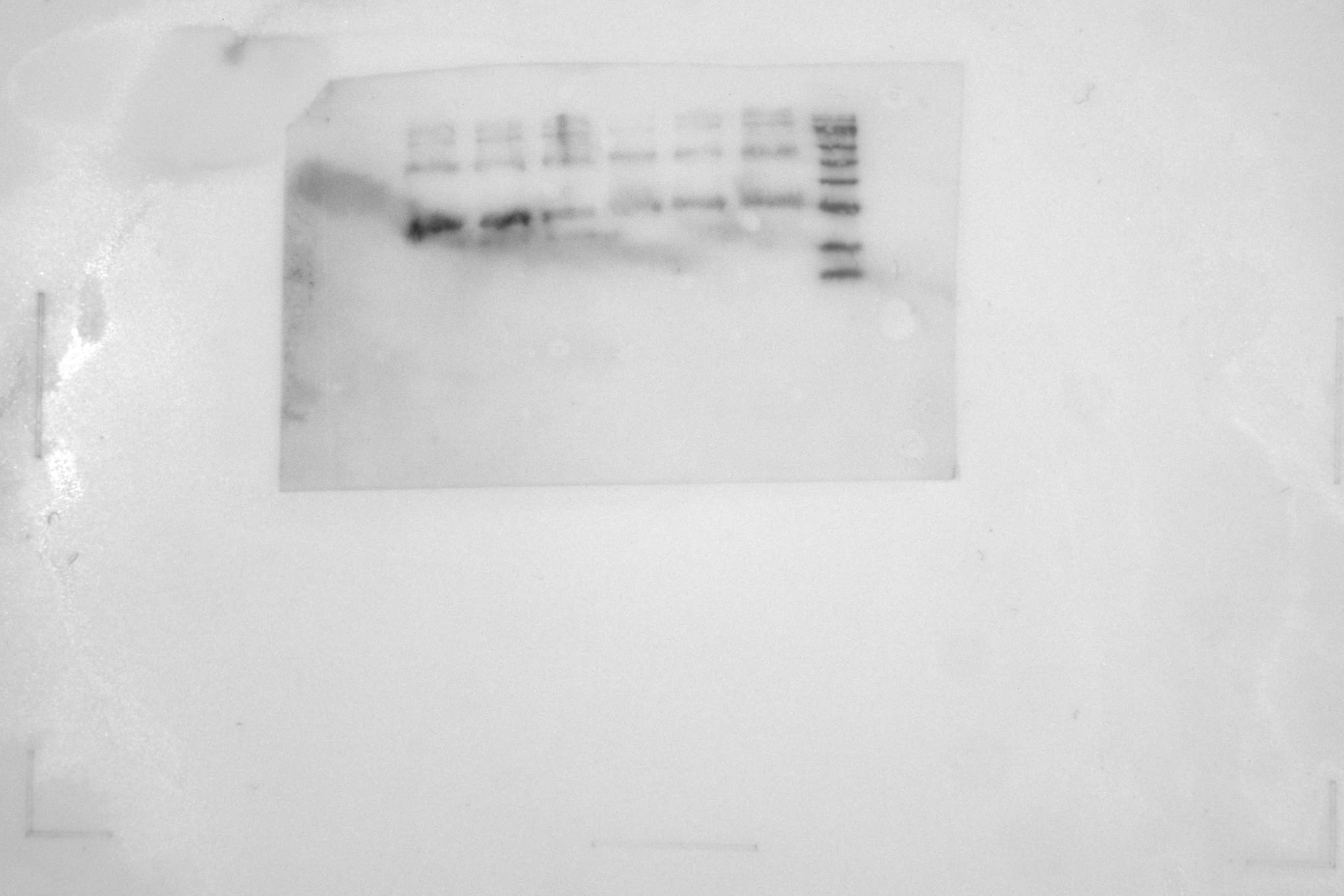

Supplement: Supplementary file 1 [file biomolecules-15-01617-s001.zip › biomolecules-3957884 - WB Original Images/Origin WB image/original WB images of Figure 4A upper.tif]
